# Detection of Drug Resistance Gene in Cutaneous Leishmaniasis by PCR in Some Endemic Areas of Iran

**Published:** 2011-12-01

**Authors:** R Alizadeh, H Hooshyar, M Bandehpor, M Arbabi, F Kazemi, A Talari, B Kazemi

**Affiliations:** 1 Department of Parasitology, School of Medicine, Kashan University of Medical Sciences, Kashan, Iran; 2 Cellular and Molecular Biology Research Center, Shaheed Beheshti University of Medical Sciences, Tehran, Iran

**Keywords:** Cutaneous Leishmaniasis, Drug resistance, PCR, Iran

## Abstract

**Background:**

Cutaneous leishmaniasis is still a health problem in many rural and urban regions of Iran and drug resistance has emerged as a major impediment in the treatment of leishmaniasis. This study aims to determine the drug resistance gene in cutaneous leishmaniasis by PCR in some endemic areas of Iran.

**Methods:**

Ninety seven samples were collected from ulcers of leishmaniasis patients from some endemic areas of Iran. The Giemsa stained samples were examined microscopically and cultured in NNN and RPMI 1640 mediums for parasite detection. After DNA extraction, PCR was done by a pair of specific primers. For detection of mutation in DNA, first PCR products were electrophoresed on CSGE gel. The suspected samples were compared by sequencing and RFLP results were demonstrated. Comparison of DNA derived from a wild type cell and mutant cell was undertaken by CSGE and sequencing methods.

**Results:**

Among 90 isolates (92.8%) examined for detection of mutation in gene with CSGE and RFLP, 10 (11.1%) revealed a disorder in sequencing selection for unresponsive to drug.

**Conclusion:**

Drug resistance in cutaneous leishmaniasis to sodium stiboglocanat is probably due to a mutation in a genome. A field study is needed to determine the distribution of drug resistance and other gene mutations involved in unresponsiveness to drugs in leishmaniasis endemic areas of Iran.

## Introduction

Protozoan parasites are responsible for some of the most important and prevalent diseases of humans and domestic animals, threaten the lives of nearly onequarter of the human population. According to the World Health Organization (WHO) reports, a 42 fold amplify of leishmaniasis has occurred in the last 15 years.[1] The pentavalent antimonial drugs of pentostam and glucantime are the first line treatments for leishmaniasis and resistance to these drugs is considered a severe clinical problem.[2] Glucantime resistance was reported in both Leishmaina tropica and Leishmania major.[3] The antimonites, which are the clinical drugs are frequently employed against leishmaniasis since 1920s, however, antimonites, have a narrow remedial window due to their toxicity and there are additional conditions which permit the resistance of Leishmania in the vertebrate host.

In fact, WHO has pointed out that the occurrence of leishmaniasis has increased since the 1980s. This maybe due to the risk of co-infection with HIV and parasites causing visceral leishmaniasis.[4]

The lack of response of Leishmania to different drugs because of sub-optimal dosages is high and rising frequently.[5] Unresponsiveness to drug has emerged as a major problem in treatment of visceral leishmaniasis with more than 50% glucantime resistance in India.[6] Several mechanisms probably are involved in such differences such as reduced drug uptake and faster drug metabolism.

In unresponsiveness to drugs, the role of multidrug resistance (MDR) genes and their products were well established .In order to determine whether MDR gene amplification and over expression can be correlated with a multidrug resistance phenotype in parasitic protozoa such as Leishmania. Transfection experiments using the MDR gene from L. donovani and L. enriettii have demonstrated that overexpression of this gene is responsible for the MDR phenotype observed in the resistant parasites.[7]

Increased cholesterol content of plasma membranes among others has recently been shown that Pglycoprotein (PGP) over-expresses the cells transected with the MDR1 gene that is cross-resistant to the ALP imofosine.[8][9][10] PGP belongs to the ATP-binding cassette (ABC) super-family of transporters is an ATP-dependent pump that exports a wide range of hydrophobic drugs from the cell, decreasing their intra- cellular concentration and preventing their cytotoxic activity, thus conferring a multi-drug resistance (MDR) phenotype during the treatment.[8],[11]

The use of pentavalent antimonials such as meglumine antimoniate (glucantime) is still the first study drug for treatment of leishmaniasis in Iran. Failure in treatment of and decrease in efficacy of glucantime were reported by some investigators in Iran.[3],[12] The cure rate of this drug is 80-85% and recently Hadighi et al. described glucantime-resistant L. tropica isolates from cutaneous leishmaniasis (CL) patients in Iran.[13]

An understanding of the resistance mechanisms to sodium stibogluconate in leishmaniasis can help us to find strategies to pass up or conquer the problem before the wide spread use of sodium stiboglocanat for the treatment of Leishmania results in the appearance of clinical cases of resistance. The systematic analysis of drug resistance mutants is useful when trying to lineate the drug target. The objective of this study was to determine of mutation in MDR gene presence in Leishmania isolated from responsive and unresponsive cases to glucantime in cutaneous leishmaniasis patients in Yazd, Mashhad and Kashan endemic provinces.

## Materials and Methods

L. tropica and L. major were isolated from 97 skin lesions from both treated and untreated patients living in some endemic regions of Iran including Yazd, Mashhad and Kashan provinces. Some of these patients did not respond to glucantim for their skin ulcers.

The amastigot form of Leishmania lesions was transferred to NNN medium culture with a liquid phase of normal saline including antibiotics (streptomycin and penicillin).The promastigote form of cloned L. tropica and L. major strains (wild type) were grown at 28°C in RPMI 1640 customized medium supplemented with 20% heat –inactivated fetal bovine serum (FBS).

Isolates of cutaneous Leishmania were grown in RPMI1640 media for 3 weeks until the culture populations were in the log phase of growth. For DNA extraction, 50 ml of liquid phase of cultures centrifuged at 430 g for 20 min at 4°C and the resulting pellets were washed with phosphate-buffer saline (pH=7.4) and centrifuged once more at 1.730 g for 20 min at 4°C. Total nucleic acid was extracted using phenol/chloroform method.[14]

A set of oligonucleotid primer, Leish ReF2, (5 '→3' TCGACCGCGAGTGTCTCAGC) and leish ReR2 (5'→3' TGGCATAGTGCGCAAAAGTG) were designed and used for PCR amplification. This primer amplified a 356 bp piece of the MDR of leishmaniasis (Accession NO L08091). The 30 µl reaction mixture contained (final concentration) 2 µl of DNA extract as template, 3 µl of 1X PCR buffer, 1.5 µl of Mgc12, 0.5 µl of deoxynucleotide triphosphate( dNTP), 0.25 µl of Taq DNA polymerase(5U/µl; Gibco, BRL), 20.75 µl of dH2O and 2 µl of each of the forward and reverse primers.

PCR reaction was carried out with 35 cycles, denaturation at 94°C for 30 sec (3 min in cycle 1), annealing at 56°C for 30 sec and elongation at 72°C for 30 sec (5 min in cycle 30). The PCR products were subjected to electrophoresis applying 1.5% agarose gel, stained by ethidium bromide and visualized under a Tran's illuminator.

This technique was developed as a result of a study in a rapid, non-radioactive-hetero duplex-based detection method for mutation screening. The method relies on the differential migration of DNA hetero-duplexes in comparison with homo duplexes during poly-acrylamide gel electrophoresis under gently denaturing conditions. For CSGE, 10 µl of PCR product was denaturized at 98°C for 5 min and then re-annealing at 68°C for 30 sec. A total of 10 µl of product and 5 µl of loading buffer were separated by electrophoresis in 10% poly-acryl-amide gel. Ethidium bromide staining and visualization under ultraviolet light determined those samples with peculiar banding patterns resulting from hetero-duplexes.

This method was used for identification of mutation. In mutant isolates, the PCR product was digested in tow band whereas in mutation region, the PCR product remained undigested. Twenty µl of PCR amplified product was digested by SDU1 restriction enzyme for 60 min at 37°C. This enzyme was selected as DNAsis software. Then it was electrophoresed on 1% agarose gel, stained by ethidium bromide and the gels were observed under ultraviolet (UV) trans-illuminator.

## Results

**Fig. 1:**
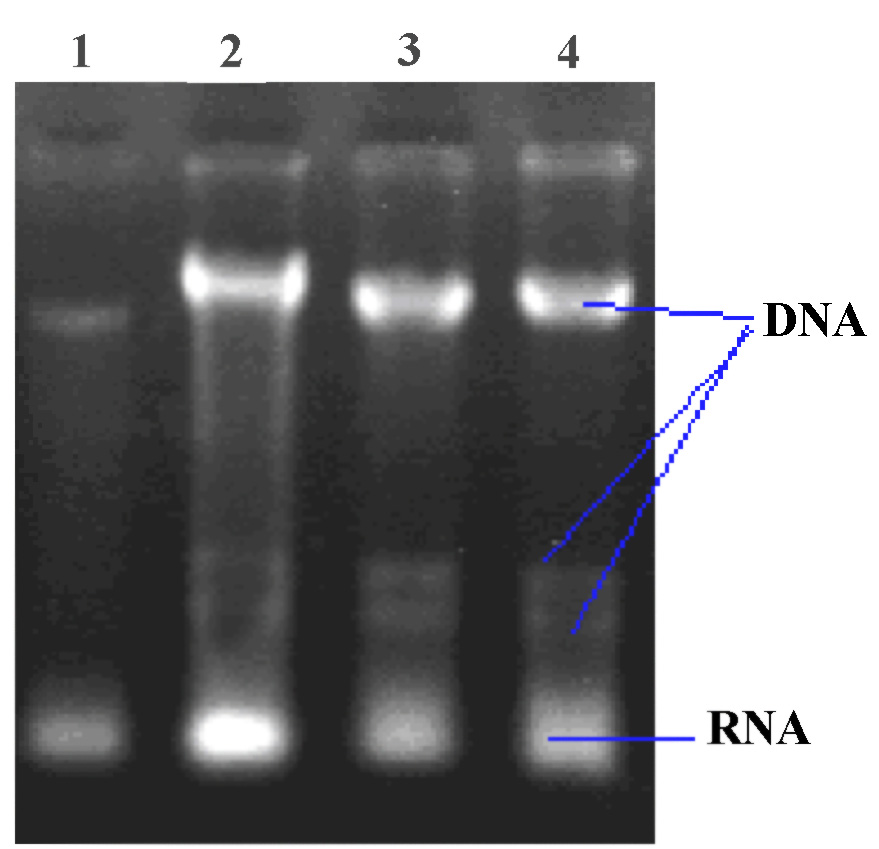
DNA extraction with phenol chloroform method.

**Fig. 2:**
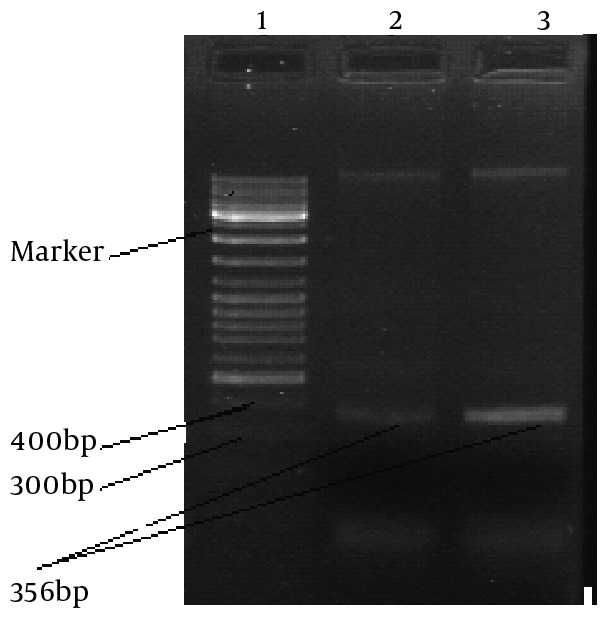
Detection of gene amplification. Ethidium bromide stained agarose gel containing molecular weight markers (line 1) and the 356 bp amplified band in line 2 and 3.

**Fig. 3:**
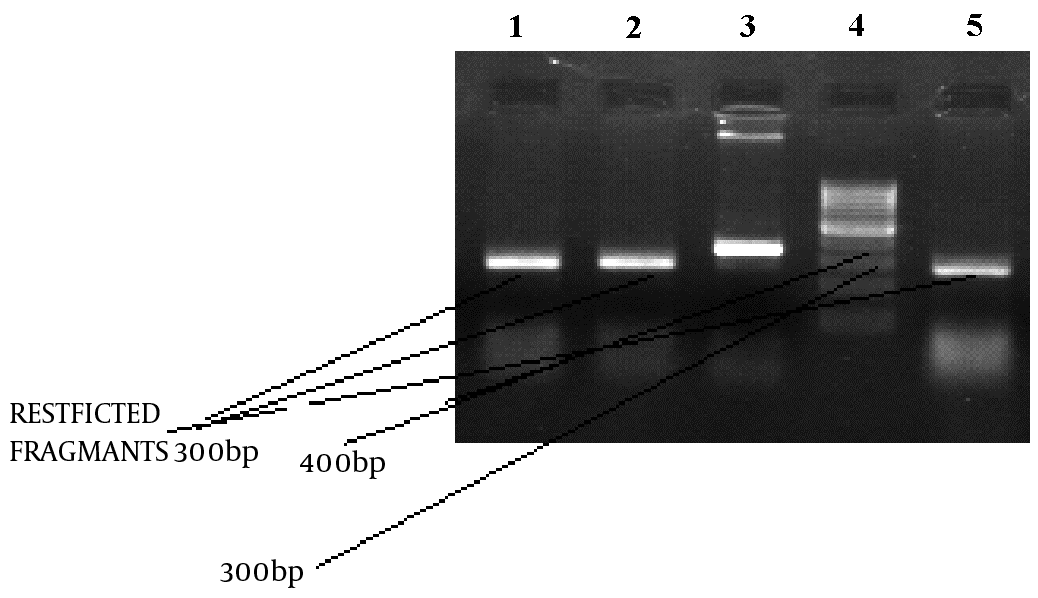
Mutation detection by use of restriction fragment length polymorphisms (RFLPs). Line 1, 2, 5 digested product; Line 3 undigested product; Line4 100 bp ladder.

A descriptive study of 97 samples of Leishmania which were collected from ulcers of patients, were observed microscopically and cultured in NNN and RPMI 1640 mediums. Genomic DNA’s were extracted from promastigote stages according to Seabrook's methods (Figure 1). By molecular examination, 90 (92.8%) samples successfully showed the desired band in PCR reaction and were examined for detection of mutation in the gene with CSGE and RFLP (Figure 2 and Figure 3).

Totally 10 samples In RFLP and CSGE presented mutation. At the end, 10 (11.1%) samples revealed a disorder in sequence selection for drug resistance (Figure 3). In resistance sample 6, samples were L. major and others were L. tropica. The mutant band were sent for sequencing and 2 patterns of nucleotide sequences of L. major were submitted to Genbank and are now available under the accession no. EU221237, EU221236.

## Discussion

Leishmaniasis is still considered a health problem in many provinces of Iran[15][16][17] while drug resistance is the other problem in treatment of diseases. Multidrug resistance-like genes have now been identified in P. falciparum, L. tarentolae and Entamoeba histolytica. [18] The aim of this study was to detect the mutations in MDR gene of agent of cutaneous leishmaniasis isolated from Iranian patients.

Sb[5] is the drug of choice against Leishmania and resistance to it has unfortunately been slow to arise. This is consistent with the hypothesis that several mutations are required for the emergence of resistance.[19] Some investigators have selected Leishmania mutants for studies of resistance to antimonial drugs.[20] Several genes are involved in resistance which are consistent with the step by step selection procedure used to generate the mutants.[21]

As in the case of tumor cells, drug resistance in Leishmania has been frequently associated with decreased cellular amassing of the drugs. Several hypotheses for decreased drug accumulation in resistant parasites have been proposed, including one analogous of the drug efflux pump model of drug resistant tumor cells, the increased expression of conserved type of protein called P-glycoprotein (p-gp) that has been one of the most consistent changes detected in drugresistant tumor cells.[22] The first MDR homologous gene discovered in Leishmania, LTPGPA, was an extra chromosomal circle (H-circle) amplified in a methotrexate resistant L. tarentolae promastigote cell line.

Since then, a group of genes belonging to the p-gp gene family and whose gene products conferred low levels of resistance to vinablastine arsenate and trivalent antimonial have been characterized in L .major. We showed that sodium stiboglocanat resistant was found in L. tropica and L. major in some endemic areas of Iran. The mutant isolates were identical to isolates with failure in therapy in clinic. It is necessary to investigate on useful alternatives drugs for treatment of leishmaniasis in resistant patients.

This study showed that unresponsiveness of cutaneus leishmaniasis to sodium stiboglocanat is due to a mutation in a genome. This mutation was seen in 10% of sample isolates. We recommend a field study to determine the distribution of drug resistance and other gene mutations involved in unresponsiveness to drug in leishmaniasis endemic areas of Iran.

## References

[1] Pérez-Victoria JM, Pérez-Victoria FJ, Parodi-Talice A, Jiménez IA, Ravelo AG, Castanys S, Gamarro F (2001). Alkyl-lysophospholipid resistance in multidrug-resistant Leishmania tropica and chemosensitization by a novel P-glycoprotein-like transporter modulator. Antimicrob Agents Chemother.

[2] Sundar S, More DK, Singh MK, Singh VP, Sharma S, Makharia A, Kumar PC, Murray HW (2000). Failure of pentavalent antimony in visceral leishmaniasis in India: report from the center of the Indian epidemic. Clin Infect Dis.

[3] Momeni A, Nadim A, Javadian E, Mohebali M, Momeni A (2009). Treatment of cutaneous leishmaniasis. Leishmaniose and Leishmania parasite 3rd Ed.

[4] Ponte-Sucre A (2003). Physiological consequences of drug resistance in Leishmania and their relevance for chemotherapy. Kinetoplastid Biol Dis.

[5] Ullman B (1995). Multidrug resistance and P-glycoproteins in parasitic protozoa. J Bioenerg Biomembr.

[6] Enk CD, Fritsch C, Jonas F, Nasereddin A, Ingber A, Jaffe CL, Ruzicka T (2003). Treatment of cutaneous leishmaniasis with photodynamic therapy. Arch Dermatol.

[7] Hendrickson N, Sifri CD, Henderson DM, Allen T, Wirth DF, Ullman B (1993). Molecular characterization of the ldmdr1 multidrug resistance gene from Leishmania donovani. Mol Biochem Parasitol.

[8] Berman JD (1997). Human leishmaniasis: clinical, diagnostic, and chemotherapeutic developments in the last 10 years. Clin Infect Dis.

[9] Brochu C, Wang J, Roy G, Messier N, Wang XY, Saravia NG, Ouellette M (2003). Antimony uptake systems in the protozoan parasite Leishmania and accumulation differences in antimonyresistant parasites. Antimicrob Agents Chemother.

[10] Bern C, Chowdhury R (2006). The epidemiology of visceral leishmaniasis in Bangladesh: prospects for improved control. Indian J Med Res.

[11] DosReis GA (2000). Susceptible hosts: a resort for parasites right in the eye of the immune response. An Acad Bras Cienc.

[12] Hadighi R, Boucher P, Khamesipour A, Meamar AR, Roy G, Ouellette M, Mohebali M (2007). Glucantime-resistant Leishmania tropica isolated from Iranian patients with cutaneous leishmaniasis are sensitive to alternative antileishmania drugs. Parasitol Res.

[13] Hadighi R, Mohebali M, Boucher P, Hajjaran H, Khamesipour A, Ouellette M (2006). Unresponsiveness to Glucantime treatment in Iranian cutaneous leishmaniasis due to drugresistant Leishmania tropica parasites. PLoS Med.

[14] Sam brooks J, Fritsch F, Maiatis T (1989). Molecular cloning: A laboratory manual, 2nd ed.

[15] Mehrabani D, Motazedian Mh, Asgari Q, Hatam GR, Owji SM, Oryan A (2011). Leishmania major in Tatera indica in Estahban, southern Iran: Microscopy, culture, isoenzyme, and PCR. Pak J Med Sci.

[16] Mehrabani D, Motazedian MH, Hatam GR, Asgari Q, Owji SM, Oryan A (2011). Leishmania major in Tatera indica in Fasa, southern Iran: Microscopy, culture, isoenzyme, PCR and morphologic study. AJAVA.

[17] Mehrabani D, Motazedian MH, Oryan A, Asqari Q, Hatam GR (2007). A search for the rodent hosts of Leishmania major in the Larestan region of southern Iran: Demonstration of the parasite in Tatera indica and Gerbillus sp., by microscopy, culture and PCR. Ann Trop Med Parasitol.

[18] Henderson DM, Sifri CD, Rodgers M, Wirth DF, Hendrickson N, Ullman B (1992). Multidrug resistance in Leishmania donovani is conferred by amplification of a gene homologous to the mammalian mdr1 gene. Mol Cell Biol.

[19] Légaré D, Hettema E, Ouellette M (1994). The P-glycoprotein-related gene family in Leishmania. Mol Biochem Parasitol.

[20] Ephros M, Waldman E, Zilberstein D (1997). Pentostam induces resistance to antimony and the preservative chlorocresol in Leishmania donovani promastigotes and axenically grown amastigotes. Antimicrob Agents Chemother.

[21] Ouellette M, Légaré D, Haimeur A, Grondin K, Roy G, Brochu C, Papadopoulou B (1998). ABC transporters in Leishmania and their role in drug resistance. Drug Resist Updat.

[22] Ouellette M, Borst P (1991). Drug resistance and P-glycoprotein gene amplification in the protozoan parasite Leishmania. Res Microbiol.

